# Modeling a role of field of view in the extraction of geometric cues during reorientation

**DOI:** 10.3389/fpsyg.2014.00535

**Published:** 2014-06-03

**Authors:** Bradley R. Sturz

**Affiliations:** Department of Psychology, Georgia Southern UniversityStatesboro, GA, USA

**Keywords:** field of view, orientation, geometry, geometric cues, theoretical model

Recently, Sturz et al. (2013) provided evidence for a role of field of view (FOV, i.e., amount of the environment visible from a single vantage point) in the reorientation process. Specifically, they found that constraining FOV (i.e., FOV of 50° compared to FOV of 100°) appeared to prevent the extraction of geometric cues. Given this evidence, I attempted to model a role of FOV in the extraction of geometric cues during reorientation. The development of such a model appeared ideal for at least two reasons. First, global geometric cues (e.g., the principal axis of space) must be, by definition, extracted from the entire environment (i.e., 100% of the environment). As a result, I assumed that 100% of the environment was the threshold for extracting global geometric cues. It follows that an inability to have available (either via current view or in memory) the entire environment would necessarily prohibit extraction of global geometric cues. However, I explicitly acknowledge that an empirical threshold for the extraction of global geometric cues is unknown. Second, recent theoretical and empirical efforts have urged for the consideration of the participant's perspective during reorientation—that is, what information is available to the perceiver (e.g., Sturz and Bodily, 2011; Wystrach and Graham, 2012). Should a model be capable of demonstrating that differences in FOV produce differences in the total amount of the environment available, it would provide additional theoretical support for a role of the participant's perspective during reorientation while also providing additional support for a role of FOV in the reorientation process. In other words, it would provide a theoretical demonstration that differences in the amount of the environment available from a single vantage point are able to produce difference in the total amount of the environment that is available to the perceiver, and by extension, delineate FOVs that would not only allow but also prohibit the extraction of global geometric cues during reorientation.

Given a fixed view at the centroid of a circular enclosure, rotating in place to view 360° of the environment is equivalent to viewing 100% of the environment. As such, an FOV of 50° would allow for viewing 13.89% whereas a FOV of 100° would allow for viewing 27.78% of the environment at any point during rotation [(50°/360°) and (100°/360°), respectively]. To illustrate how these differences in the amount of the environment viewable from a single vantage point would translate into time course differences in the total amount of the environment available, Figure 1 illustrates (A) and plots (B) the percent of the environment available by time in the environment for different FOVs. For this initial model, I assumed a fixed viewpoint from the centroid of a circular enclosure at a fixed rate of rotation (either clockwise or counter-clockwise—shown clockwise in top panel) of 50° every 1.0 s coupled with no loss in memory across time. Such a model can be expressed as a linear function:

(1) f(x)=a+bx

Where *a* = percent of environment of current view, *b* = rate of rotation, and *x* = time in the environment.

**Figure 1:**
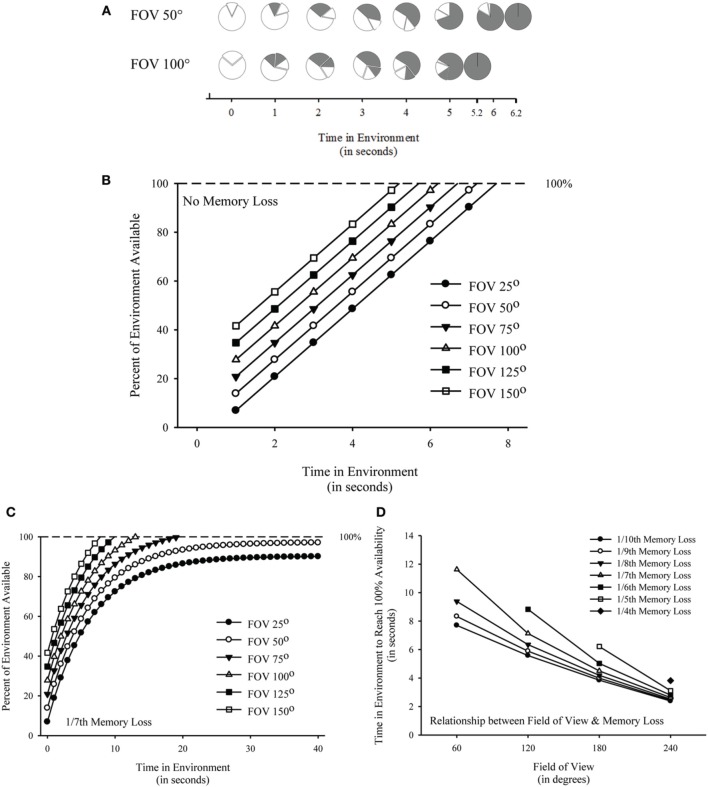
**(A)** Theoretical illustration of percent of environment available by time in the environment assuming a fixed field of view at the centroid of a circular enclosure with a constant rate of rotation (i.e., 50° clockwise every 1.0 s) and no loss in memory plotted for a field of view of 50° and a field of view of 100°. Exploded regions represent the current field of view. Filled regions represent the amount of environment previously viewed. Unfilled regions represent the amount of the environment that is not yet viewed. Theoretical model of percent of environment available by time in the environment assuming a fixed field of view at the centroid of a circular enclosure with a constant rate of rotation (i.e., 50° every 1.0 s) and no memory loss **(B)** or 1/7th loss of memory every 1.0 s **(C)** plotted for a field of view of 25, 50, 75, 100, 125, and 150°. **(D)** Time in environment to reach 100% availability plotted by field of view for memory loss values of 1/10, 1/9, 1/8, 1/7, 1/6, 1/5, and 1/4th.

As shown in Figure 1B, all six FOVs would eventually reach 100% availability of the environment but differ with respect to time course; however, all FOVs would be able to extract global geometric cues from the environment because all would cross the threshold for having the entire environment available (i.e., 100%). As a result, this model fails to capture a condition under which the extraction of global geometric cues would be prevented for some FOVs but not others. Despite this limitation, it is worth noting that this model demonstrates that the length of time to have the entire environment available would differ across manipulations of FOV. It is also important to note that this model was based on an assumption of no loss in memory across time; as a result, the model fails to incorporate the well-established fact that there is memory loss across time (for a review, see Neath and Surprenant, 2003; see also Allen, 2004).

I modified the initial model by including a modest rate of memory loss (1/7th lost every 1.0 s). Such a model can be expressed as an exponential function:

(2) $$f(x)=a+[7b-(a*c)]*(1-d^{x})$$

Where *a* = percent of environment of current view, *b* = rate of rotation, *c* = proportion of current view that is novel (i.e., 1.0 for each rotation for an FOV of 50° and 0.5 for each rotation for an FOV of 100°), *d* = proportion of memory remaining (i.e., 6/7 = ~0.86), and *x* = time in the environment.

As shown in Figure 1C, it becomes apparent that some FOVs (e.g., 75, 100, 125, and 150°) will result in an ability to have the entire environment available (i.e., above the 100% threshold for extraction of global geometric cues) whereas other FOVs (e.g., 25 and 50°) will fail to have the entire environment available (reaching asymptotic levels below the 100% threshold for extraction of global geometric cues). As a result, incorporating a low rate of memory loss is able to capture a difference in the amount of the environment that is available between different FOVs. It is worth noting that this model demonstrates an inability of an FOV of 25 and 50° to have the entire environment available.

In an effort to model the relationship between FOV and amount of memory loss (both of which may differ across species), I solved for the amount of time required in the environment to reach 100% availability of the environment (i.e., x in Function 2) using the equation:

(3) $$x=-1*\;\left[log_{d}*\left(\frac{100-a}{[(Constant*b)-(a*c)]-1}\right)\right]$$

Figure 1D plots FOV (60, 120, 180, and 240°) by time to have available 100% of the environment for various memory loss values (1/10, 1/9, 1/8, 1/7, 1/6, 1/5, 1/4th) all with a 60° rate of rotation. As shown, time to reach 100% decreased with increased FOVs, but time to reach 100% increased with increased amount of memory loss. Importantly, some combinations of FOV and memory loss prohibit reaching 100% availability of the environment (e.g., FOV 60° with 1/6th memory loss).

These relatively simple models establish that the amount of the environment visible from a single vantage point is theoretically capable of producing differences in the total amount of the environment available to participants and establish conditions under which FOV can prohibit and allow the extraction of global geometric cues. I explicitly acknowledge the limitations of the models' assumptions^1^ (e.g., the rate of rotation which may increase linearly with FOV, the amount of memory loss, the threshold for extraction of geometric properties and relationships of space) along with the omission of additional model parameters (such as the shape of the environment, movement within the environment, variable locations within the environment, differences in the amount of the environment viewed across rotations, and back rotations of views within the environment). Despite these limitations, the above models appear to incorporate minimal and fundamental parameters while providing additional support for the consideration of the participant's perspective, establishing a theoretical possibility for a role of FOV in the extraction of global geometric cues during reorientation, and delineating theoretical predications against which empirical results could be compared.

## Conflict of interest statement

The author declares that the research was conducted in the absence of any commercial or financial relationships that could be construed as a potential conflict of interest.

## Footnotes

^1^An Excel file is available for download at https://sites.google.com/a/georgiasouthern.edu/bradley-r-sturz/FOV_Model_Functions.xlsx which allows users to modify model parameters such as field of view, rate of rotation, and amount of memory loss to determine their influence on the amount of environment that is available across time in the environment.
